# Misoprostol administered sublingually at a dose of 12.5 μg versus vaginally at a dose of 25 μg for the induction of full-term labor: a randomized controlled trial

**DOI:** 10.1186/s12978-020-0901-8

**Published:** 2020-04-10

**Authors:** Daniele S. M. B. Gattás, Melania M. R. de Amorim, Francisco E. L. Feitosa, José R. da Silva-Junior, Lívia C. G. Ribeiro, Gustavo F. A. Souza, Alex S. R. Souza

**Affiliations:** 1 Postgraduate Program in Comprehensive Healthcare at the Instituto de Medicina Integral Prof. Fernando Figueira (IMIP), Rua Dom Sebastião Leme 171/ 2702, Graças, Recife, Pernambuco 52011-160 Brazil; 2 Department of Obstetrics and Gynecology, Federal University of Campina Grande (UFCG) and Instituto de Pesquisa Professor Joaquim Amorim Neto (IPESq), Campina Grande, Paraíba Brazil; 3 Assis Chateaubriand Maternity Teaching Hospital, Federal University of Ceará (UFC), Fortaleza, Ceará Brazil; 4 Department of Obstetrics and Gynecology, Faculdade Pernambucana de Saúde (FPS), Recife, Pernambuco Brazil; 5 Undergraduate medical student, Centre for Biological Sciences and Health, Catholic University of Pernambuco (UNICAP), Recife, Pernambuco Brazil; 6 Postgraduate Program in Comprehensive Healthcare, Instituto de Medicina Integral Prof. Fernando Figueira (IMIP), Department of Maternal and Child Health, Federal University of Pernambuco (UFPE), Centre for Biological Sciences and Health, Catholic University of Pernambuco (UNICAP), Recife, Pernambuco Brazil

**Keywords:** Administration, sublingual, Clinical trial, Labor, induced, Labor, obstetric, Misoprostol/administration & dosage, Multicenter study

## Abstract

**Background:**

Labor induction is defined as any procedure that stimulates uterine contractions before labor begins spontaneously. The vaginal and oral routes of administration of misoprostol are those most used for the induction of labor in routine practice, with the recommended dose being 25 μg. Nevertheless, the sublingual route may reduce the number of vaginal examinations required, increasing patient comfort and lowering the risk of maternal and fetal infection. Based on a previous systematic review, the objective of this study was to compare the frequency of tachysystole as the main outcome measure when misoprostol is administered sublingually at the dose of 12.5 μg versus vaginally at a dose of 25 μg to induce labor in a full-term pregnancy with a live fetus.

**Methods:**

A randomized, placebo-controlled, triple-blind clinical trial was conducted at two maternity hospitals in northeastern Brazil. Two hundred patients with a full-term pregnancy, a live fetus, Bishop score ≤ 6 and an indication for induction of labor were included. Following randomization, one group received 12.5 μg misoprostol sublingually and a vaginal placebo, while the other group received a sublingual placebo and 25 μg misoprostol vaginally. The primary outcome was the frequency of tachysystole. Student’s t-test, the chi-square test of association and Fisher’s exact test were used, as appropriate. Risk ratios and their 95% confidence intervals were calculated.

**Results:**

The frequency of tachysystole was lower in the group using 12.5 μg misoprostol sublingually compared to the group using 25 μg misoprostol vaginally (RR = 0.15; 95%CI: 0.02–0.97; *p* = 0.002). Failure to achieve vaginal delivery within 12 and 24 h was similar in both groups. Sublingual administration was preferred to vaginal administration by women in both groups; however, the difference was not statistically significant.

**Conclusion:**

The effectiveness of labor induction with low-dose sublingual misoprostol was similar to that achieved with vaginal administration of the recommended dose; however, the rate of tachysystole was lower in the sublingual group, and this route of administration may prove a safe alternative.

**Trial registration:**

Registration number: NCT01406392, ClinicalTrials.gov. Date of registration: August 1, 2011.

## Plain English summary

Labor induction refers to any procedure used to stimulate uterine contractions before labor begins spontaneously. The drug misoprostol, used at a dose of 25 μg, can encourage labor to begin and is commonly given to women by mouth (oral administration) or by placing the tablet in the woman’s vagina (vaginal administration). When used under the tongue (sublingual administration), misoprostol has the advantage of reducing the need for numerous vaginal examinations, increasing the woman’s comfort and decreasing the risk of infection for mother and child. We compared two groups of women with a full-term pregnancy and a live fetus. Labor induction was indicated in all cases to allow vaginal delivery to occur. In the first group, a low-dose tablet of misoprostol (12.5 μg) was placed under the pregnant woman’s tongue and a placebo tablet was inserted into her vagina at the same time, while in the second group a tablet with the usual dose of misoprostol (25 μg) was placed in the woman’s vagina and a placebo tablet under her tongue also at the same time. In the first group, fewer women developed excessively frequent uterine contractions (known as tachysystole) compared to the second group. The number of women unable to give birth vaginally (women who ultimately needed a caesarian section) was similar in the two groups. We concluded that low-dose tablets of misoprostol given sublingually to induce labor were as effective as the recommended dose administered vaginally, but with the advantage of causing fewer cases of tachysystole. Other studies with larger numbers of women are necessary to confirm the safety of low-dose tablets of misoprostol given sublingually.

## Background

Labor induction is defined as any procedure that stimulates uterine contractions before labor begins spontaneously [1]. This is an option when the goal is vaginal birth and the risk for the mother and child of continuing the pregnancy exceeds the risk of interrupting it [2]. Labor induction is indeed common, being required in a quarter of all high-risk pregnancies and in one-tenth of normal-risk pregnancies [3]. Despite great efforts to identify an optimal method, up to now no protocol for labor induction has been found to be completely risk-free [4].

Misoprostol is a synthetic analogue of prostaglandin E1 that acts on the cervix and uterine smooth muscle, facilitating dilatation and promoting uterine contractions [5]. It has been compared to other methods for inducing labor, and different routes of administration and dosage regimens have been evaluated [6].

The vaginal and oral routes of misoprostol administration are those most used to induce labor in routine practice, with the recommended dose being 25 μg [7]. Nevertheless, the sublingual route [8] may reduce the number of vaginal examinations required, increasing patient comfort and lowering the risk of maternal and fetal infection [9].

A systematic review involving five high-quality clinical trials (*n* = 740) compared sublingual misoprostol and vaginal misoprostol at different doses to induce labor in women bearing a live, full-term fetus. No significant differences were found between the two routes of administration in relation to the frequency of vaginal delivery at 24 h, uterine hyperstimulation or Caesarean section. Nevertheless, an increased risk of tachysystole was found in the sublingual group, an effect that was probably dose-dependent. Therefore, although sublingual misoprostol was effective in inducing labor, further studies were recommended to determine its safety [10].

In a pilot study with low dose (12.5 μg) sublingual misoprostol, labor was successfully triggered in 90% of cases, with 60% progressing to vaginal delivery, 47% of these within the first 24 h. The frequency of tachysystole was 6.7% [11], lower than that of 11.4% reported in the meta-analysis [10].

In view of the limitations of a single study with a low dose of sublingual misoprostol, the present study aimed to compare the frequency of tachysystole using 12.5 μg of misoprostol sublingually with 25 μg (the recommended dose) of the drug used vaginally to induce labor in women bearing a live, full-term fetus.

## Methods

A controlled, randomized, triple-blind clinical trial was conducted with 200 pregnant women with an indication for induction of labor at the *Instituto de Medicina Integral Prof. Fernando Figueira* (IMIP), Recife, Pernambuco, and at the Assis Chateaubriand Maternity Teaching Hospital (MEAC) of the Federal University of Ceará, Fortaleza, Ceará, both in northeastern Brazil, between July 2014 and November 2016. The study was registered at clinicaltrials.gov under reference NCT01406392.

Sample size was calculated using the Statcalc tool of Epi Info, version 3.5.2 for Windows (Centers for Disease Control and Prevention [CDC], Atlanta, GA, USA). Predicting a frequency of tachysystole of 6.7% in the group of women using 12.5 μg misoprostol sublingually [11] and of 21.6% in the group using 25 μg of misoprostol vaginally [5], for a 95% confidence level and a power of 80%, 98 women would have to be recruited to each group. To compensate for any possible losses, the total sample size was increased to 200 participants, 100 in each group.

The inclusion criteria were: indication for induction of labor, gestational age ≥ 37 weeks, single live fetus, vertex presentation, Bishop score ≤ 6, estimated fetal weight < 4000 g, amniotic fluid index > 5 and good fetal well-being. The exclusion criteria were: previous Caesarean section, previous uterine scar resulting from uterine surgery, genital bleeding of unknown origin, fetal abnormalities, chorioamnionitis, tumors, malformations and/or ulceration in the vulvar or perineal region or birth canal that could be harmful to mother or child during the expulsion stage of labor, and HIV positivity. Eligibility was determined by performing digital vaginal examination to evaluate the Bishop score [12]; rapid HIV testing; and ultrasonography to estimate fetal weight and measure amniotic fluid volume. In addition, fetal well-being was assessed using Doppler flow velocity and/or cardiotocography and/or fetal biophysical profile and/or fetal vibroacoustic stimulation, depending on what was available in the maternity hospitals.

Doctors on duty in the Obstetrics Departments of both participating institutions referred potential candidates to the study investigators. All the women who fulfilled the eligibility criteria received explanations on the relevance of conducting the study, the study objectives and the possible consequences of their participation. Women were assured that should they not wish to participate in the study they would continue to receive care from a trained medical team in accordance with the routine practice at the hospital. Potential volunteers were given time to read the informed consent form carefully and had the opportunity to ask questions. Only the women who signed the informed consent form were admitted to the study. An independent data safety monitoring board supervised the occurrence of adverse events and had the power to require an interim analysis or break the blinding code at any moment if considered necessary.

The vaginal and sublingual tablets of misoprostol and placebo were manufactured by *Laboratório Hebron S.A. Indústrias Químicas e Farmacêuticas* (Caruarú, Pernambuco, Brazil). Misoprostol for vaginal use consists of misoprostol together with lactose, microcrystalline cellulose, aerosil, explocel and sorbitol, and is available commercially under the trade name Prostokos® (25 μg). For this study, the pharmaceutical company also prepared the drug for sublingual use at the dose of 12.5 μg of misoprostol together with spray-dried lactose, croscarmellose sodium, crospovidone and magnesium stearate.

Randomization was performed using a single block of sequential numbers from 1 to 200 and the letters A and B. A statistician not otherwise involved in the study and who was unaware of what A and B represented prepared the list using the Random Allocation software program, version 1.0 (Isfahan, Iran). This list was sent to *Laboratório Hebron* where the pharmacist responsible for preparing the medication defined the meaning of A and B (sublingual or vaginal administration), without the investigators or the statistician having access to this information. Standardized, sequentially numbered, identical opaque packages were prepared in accordance with the randomization list. Each package contained either 8 active sublingual tablets of misoprostol (12.5 μg) and 8 placebo vaginal tablets or 8 placebo sublingual tablets and 8 active vaginal tablets of misoprostol (25 μg). Information on the contents of each package remained concealed until data analysis was complete. The vaginal and sublingual placebo tablets were identical in shape, size, color, smell, taste and weight to the tablets containing the active substance and were specially prepared for this study. Therefore, the triple-blind procedures were assured, since neither the investigators nor the patients nor the statistician were aware of the contents of each package.

To induce labor, the attending physician administered the sublingual tablets (misoprostol or placebo) and the vaginal tablets (misoprostol or placebo) at the same time, every 6 h, except when the mother was asleep, up to a maximum of eight tablets. Whenever possible, the induction process began at 6 am and was interrupted at 10 pm if labor had not begun in the first 12 h. The sublingual tablets were placed under the tongue and the vaginal tablets were inserted into the posterior fornix, with the patient then being instructed to lie on her left side for 1 h to allow the tablet to dissolve spontaneously.

Patient monitoring and care occurred with no interference whatsoever from the investigators. The patients were examined every 30 min to evaluate fetal heart rate (FHR) and uterine dynamics. Digital vaginal examination was only performed to re-evaluate the Bishop score when the vaginal medication was given or when labor began or 6 h after administration of the final tablet in order to diagnose failed induction of labor.

If any change in uterine contractility such as tachysystole, hypertonus or uterine hyperstimulation was identified during monitoring, the patient was placed in the left lateral decubitus position and hydrated with 1000 ml of Ringer’s lactate solution in 30 min, and oxygen therapy and tocolysis with oral nifedipine 10 mg were prescribed [13]. If the pattern of contractions and/or FHR did not return to normal, Caesarean section was indicated. Induction of labor was considered to have failed if labor had not been triggered 6 h after administration of the final tablet, and a Caesarean section was then performed.

The primary outcome was the frequency of tachysystole. Secondary outcomes were: changes in the cervix at 12 and 24 h; failure to achieve vaginal delivery within 12 and 24 h; the mother’s preferred route of administration; time between the first dose and the onset of labor and delivery; duration of labor, need for oxytocin; failed induction of labor; Caesarean section and its indications; uterine hyperstimulation, need for epidural anesthesia; maternal side effects (nausea, vomiting, diarrhea, postpartum hemorrhage, fever); severe maternal morbidity (uterine rupture, sepsis, admission to intensive care unit) or maternal death; meconium in the amniotic fluid; non-reassuring FHR; one- and five-minute Apgar scores < 7, admission of the newborn to a neonatal intensive care unit; need for neonatal resuscitation; and severe neonatal morbidity (convulsions and neonatal asphyxiation) or perinatal death.

The control variables were mother’s age; gestational age at admission; amniotic fluid index; estimated fetal weight; parity; Bishop score; and the indications for induction of labor.

The abnormalities in uterine contractility evaluated were tachysystole (the presence of ≥6 uterine contractions for two consecutive 10-min periods) [14, 15]; uterine hypertonus (a single contraction lasting 2 min or longer); and uterine hyperstimulation, with the presence of tachysystole or uterine hypertonus associated with non-reassuring FHR [14–16]. Non-reassuring FHR was defined as the persistence of FHR < 110 bpm or late decelerations in FHR (reduction in FHR following a uterine contraction - type 2 dip) and/or fetal tachycardia, persistent FHR > 160 bpm [16].

Statistical analysis was conducted using Epi-Info, version 3.5.3 (CDC, Atlanta, GA, USA). The categorical variables were compared using the chi-square test of association or Fisher’s exact test, as appropriate. Student’s t-test was used to compare the continuous variables with normal distribution and variances, while the non-parametric Mann-Whitney test was used for the discrete, ordinal or continuous variables for which distribution was not normal. *P*-values were two-tailed for all tests and the significance level adopted was 5%.

Risk ratios (RR) and their 95% confidence intervals (95%CI) were calculated as a measure of relative risk. The number needed to treat (NNT) and the number needed to harm (NNH) were calculated for the primary endpoint.

## Results

**Fig. 1:**
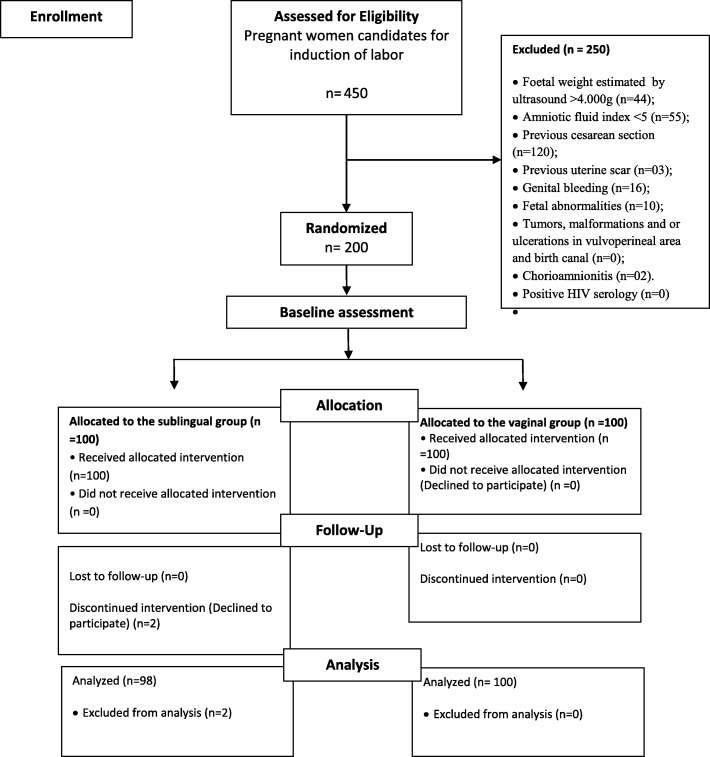
Flow diagram of procedures for selection and monitoring of subjects (CONSORT)

Of the 450 women eligible for the study, 250 were excluded for the following reasons: 120 had had a previous Caesarean section; in 44 cases estimated fetal weight was > 4.000 kg; in 55 cases, the amniotic fluid index was < 5.0 cm; 3 women had uterine scars from previous uterine surgery; 10 had an ultrasonographic diagnosis of fetal abnormalities; 16 had genital bleeding of unknown origin; and 2 had chorioamnionitis. A further two women in the sublingual group refused to continue in the study (Fig. 1); therefore, 198 women were included in the final analysis.

The maternal and obstetric characteristics of both groups were similar (Table 1). The principal indications for induction of labor were hypertensive pregnancy disorders, prolonged pregnancy, diabetes and premature rupture of membranes. The frequency of hypertensive disorders was higher in the sublingual group (54.1% versus 32.0%, RR, 1.56, 95%CI: 1.18–2.07; *p* = 0.002) (Table 2).

**Table 1:** Maternal and obstetric characteristics according to the route of administration of misoprostol (sublingual or vaginal)

Characteristic	Sublingual Misoprostol (***n*** = 98)		Vaginal misoprostol (***n*** = 100)	
Maternal age (years) (Mean; SD)	26.5	6.9	25.9	6.4
Gestational age at admission (weeks) (mean; SD)	38.7	1.4	39.1	1.5
Amniotic fluid index (Mean; SD)	12.2	5.1	12.1	5.5
Estimated fetal weight (grams) (mean; SD)	3235.0	392.7	3254.7	317.4
Number of previous pregnancies (Median; IQR)	1	1–2	1	1–2
Parity (Median; IQR)	0	0–1	0	0–1
Bishop score (median; IQR)	3	2–4	3	2–4

*SD* Standard deviation, *IQR* Interquartile range

**Table 2:** Indications for induction of labor according to the route of administration of misoprostol

Indications	Sublingual misoprostol (***n*** = 98)		Vaginal misoprostol (***n*** = 100)		RR	95% CI	***p***-value*
	n	%	n	%			
Hypertensive syndromes	53	54.1	32	32.0	1.56	1.18–2.07	0.002
Prolonged pregnancy	20	20.4	32	32.0	0.72	0.49–1.05	0.06
Premature rupture of membranes	9	9.2	6	6.0	1.23	0.79–1.91	0.39
Diabetes	16	16.3	25	25.0	0.75	0.49–1.13	0.13
Others	12	12.2	11	11.0	1.06	0.69–1.61	0.78

*RR* Relative risk, *CI* Confidence interval; n: sample; %: percentage. * Chi-square test

The frequency of tachysystole was lower in the low-dose sublingual group compared to the vaginal group (1.0% versus 12.0%; RR: 0.15; 95%CI: 0.02–0.97; *p* = 0.002; NNH = 9.1). The rate of failure to achieve vaginal delivery within 12 and 24 h and the secondary endpoints were similar in both groups (Table 3).

**Table 3:** Outcomes following induction of labor according to the route of administration of misoprostol

Endpoints	Sublingual misoprostol (***n*** = 98)		Vaginal misoprostol (***n*** = 100)		RR	95% CI	***p***-value
	n	%	n	%			
Tachysystole (> 6 contractions in 10 min)	1	1	12	12	0.15	0.02–0.97	0.002**
Change in cervix after 12 h	58	59.2	52	52.0	1.16	0.87–1.55	0.31**
Change in cervix after 24 h	73	74.5	74	74.0	1.01	0.73–1.40	0.94**
Failure to achieve vaginal delivery within 12 h	90	91.8	93	93.0	0.92	0.56–1.51	0.76**
Failure to achieve vaginal delivery within 24 h	70	71.4	66	66.0	1.14	0.83–1.57	0.41**
Need for oxytocin	33	33.7	24	24.0	1.25	0.94–1.67	0.13**
Epidural anesthesia	2	2.0	1	1.0	1.35	0.60–3.05	0.98*
Maternal preference for sublingual route	65	66.3	54	54.0	1.31	0.96–1.78	0.08**
Nausea	5	5.1	9	9.0	0.71	0.34–1.45	0.28**
Vomiting	2	2.0	2	2.0	1.01	0.37–2.72	1.00*
Diarrhea	1	1.0	2	2.0	0.67	0.13–3.34	1.00*
Caesarean section	56	57.1	56	56.0	1.02	0.77–1.36	0.87**
Meconium	6	6.1	11	11.0	0.69	0.36–1.34	0.22**
Admission to neonatal intensive care unit	1	1.0	1	1.0	1.01	0.25–4.07	1.00*
Neonatal resuscitation	4	4.1	2	2.0	1.36	0.76–2.44	0.66*

*CI* Confidence interval. * Fisher’s exact test, ** Chi-square test, RR: relative risk

There were no cases of maternal hyperthermia, severe maternal morbidity or maternal death and no cases of severe neonatal morbidity, perinatal death, need for mechanically assisted ventilation, neonatal encephalopathy or neonatal infection.

The mean time between the first dose of misoprostol and the onset of labor was 22.5 ± 15.1 h in the sublingual group versus 28.0 ± 17.3 h in the vaginal group (*p* = 0.06). The mean time between the first dose and delivery (39.4 ± 21.2 versus 39.9 ± 21.3 h; *p* = 0.86) and the mean duration of labor (7.3 ± 3.9 versus 8.3 ± 3.9 h; *p* = 0.15) were similar in both groups.

Median Apgar score was 8 at the first minute (*p* = 0.43) and 9 at the fifth minute (*p* = 0.36) in both groups. There was no difference in mean birthweight between the sublingual and vaginal groups (3268.3 ± 458.9 g versus 3322.1 ± 426.9 g; *p* = 0.39).

Caesarean rates were similar in both groups (Table 3), as were the indications for the procedure. The principal indication was failed induction of labor in 34.7% of patients in the sublingual group and 23.0% in the vaginal group (RR: 1.31; 95%CI: 0.99–1.74; *p* = 0.07). There were three cases (3.1%) in the sublingual group in which a Caesarean section was required due to non-reassuring FHR compared to six cases in the vaginal group (RR: 0.66; 95%CI: 0.26–1.69; *p* = 0.51). Labor dystocia was more common in the sublingual group, while cephalopelvic disproportion was more common in the vaginal group (Table 4).

**Table 4:** Indications for Caesarean section following labor induction according to the route of administration of misoprostol

Indications for Caesarean Section	Sublingual misoprostol (***n*** = 98)		Vaginal misoprostol (***n*** = 100)		RR	95% CI	***P***-value
	n	%	n	%			
Failed induction	34	34.7	23	23.0	1.31	0.99–1.74	0.07**
Labor dystocia	9	9.2	4	4.0	1.44	0.97–2.13	0.14**
Non-reassuring fetal heart rate	3	3.1	6	6.0	0.66	0.26–1.69	0.51*
Cephalopelvic disproportion	3	3.1	9	9.0	0.49	0.18–0.32	0.08**
Persistent hypertonus/hypersystole	1	1	2	2.0	0.67	0.13–3.36	1.00*
Persistent tachysystole	0	0	2	2.0	0	_____	0.51*
Others	8	8.2	11	11.0	0.84	0.48–1.45	0.49**

*RR* Relative risk, *CI* Confidence interval, n: sample, %: percentage. * Fisher’s exact test; ** Chi-square test

## Discussion

In this study, the rate of tachysystole was lower with 12.5 μg misoprostol administered sublingually for the induction of labor compared to 25 μg administered vaginally. A systematic review that included five clinical trials (*n* = 740) and compared sublingual with vaginal misoprostol found a greater risk of tachysystole in the sublingual group (OR: 1.70; 95% CI: 1.02–2.83) [10]. That effect was possibly dose-dependent, since the higher the dose of misoprostol, the higher the risk of tachysystole. The present study aimed to identify a lower sublingual dose that would prove effective in triggering labor with a minimum of side effects. Indeed, a lower rate of tachysystole was found in the sublingual group compared to the vaginal group (1.0% versus 12.0%; RR: 0.15; 95% CI: 0.02–0.97; *p* = 0.002), corroborating the previous suggestion of a dose-dependent effect.

The maternal, obstetric and neonatal characteristics evaluated were similar in both groups, confirming the homogeneity of the sample. The lower the Bishop score, the greater the risk of failed induction of labor [12]. The median Bishop score was 3 with both routes of administration, suggesting that low-dose (12.5 μg) misoprostol administered sublingually can induce labor in women with an unfavorable cervix just as well as when administered by the vaginal route at a dose of 25 μg.

Both groups were similar regarding the different secondary endpoints, suggesting that, despite the reduced dose of misoprostol used in the sublingual group, effectiveness remained the same as when a 25 μg-dose is provided vaginally. Changes in the cervix at 12 and 24 h and the rate of failure to achieve vaginal delivery within 12 and 24 h were similar in both groups. Other studies in which the vaginal and sublingual routes were compared using different doses from those used in this study reported a similar rate of effectiveness [4, 10]; however, with more side effects.

The Caesarean section rate was similar in both groups and high compared to the rate of 15% recommended by the World Health Organization [17]. This may be due to the profile of patients in these tertiary care centers, which tend to receive women with high-risk pregnancies. In agreement with another study conducted in this same population, hypertensive disorders of pregnancy were the principal indications for induction of labor in both groups (54.1% versus 32.0%) [18].

High-risk pregnancies may have affected Caesarean-section rates; however, Caesarean rates are habitually high in studies conducted in Brazil, particularly when labor is induced [18, 19]. This may also have reflected on the principal indication for Caesarean section in both groups, failed induction of labor, which differs from the findings of other studies conducted in different countries in which the principal indication for performing a Caesarean section was non-reassuring FHR [4, 20, 21].

The time between the first dose of misoprostol and the onset of labor was shorter in the sublingual group (22.5 versus 28.0 h); however, this difference was not statistically significant and may have occurred because peak drug concentration is reached in a faster time with sublingual administration than with vaginal administration, as already shown in a pharmacological study [22].

The need for oxytocin was greater, although not significantly so, in the sublingual group; however, this rate was lower than that reported by other authors [9, 21]. This greater need for oxytocin could be explained by the low dose of sublingual misoprostol administered. When labor is triggered and administration of the drug is interrupted, progression of the labor curve decelerates, and it may become necessary to initiate oxytocin to maintain the pattern of the speed of cervical dilatation and uterine contractions. This time was probably recuperated with the use of oxytocin, since the duration of labor was shorter in the sublingual group (7.3 versus 8.3 h).

The time between the first dose of the medication and the onset of labor and delivery was greater in the present study than that described by other authors, irrespective of the route of administration [9, 18, 21, 23]. This may have occurred because the protocol of the present study included a scheduled interruption in induction from 10 pm until 6 am if labor had not already been triggered. This pause was to provide greater comfort overnight to the participating women. However, in the other studies, induction of labor continued uninterrupted at night.

The principal side effects with misoprostol include changes in uterine contractility, hyperthermia, nausea, vomiting and diarrhea [24]. The number of patients with nausea, vomiting and diarrhea was lower in the sublingual group, although not significantly so. In agreement with the results of other studies, the frequency was low [4, 9, 21, 23]; nevertheless, the sample was insufficiently sized to identify differences between the groups.

There was a greater maternal preference for the sublingual route. Other authors have also reported greater maternal satisfaction with the sublingual route [9, 25] possibly due to the need for fewer digital vaginal examinations, thus providing patients with greater comfort. However, in the present study both routes of administration were used in all cases, with the active drug being administered by one route and the placebo by the other. Therefore, following childbirth, patients merely stated their preference for one route or the other.

Regarding perinatal endpoints, meconium was present in the amniotic fluid in 6.1% of cases in the sublingual group, lower than the rate of 11% found in the vaginal group. This value is also lower than rates reported in the literature [4, 20, 21]. This low frequency of meconium in the amniotic fluid may be a consequence of the low dose of medication used. The median first- and fifth-minute Apgar scores were the same in both groups and similar to those reported in a study conducted in Uruguay [26].

Misoprostol, administered sublingually at a low dose, was safe, as shown by the rate of altered uterine contractility (tachysystole), and effective, as shown by its ability to trigger labor. Further randomized studies involving low doses of misoprostol administered sublingually should be performed with larger sample sizes to determine the effectiveness and safety of this regimen, and a subsequent meta-analysis should be conducted.

The strength of this study resides in the fact that it was the first trial to compare a low dose of sublingual misoprostol (12.5 μg) with a 25-μg dose of vaginal misoprostol. The main limitation of the study was its small sample size.

## Conclusion

The effectiveness of labor induction with low-dose sublingual misoprostol was similar to that achieved with vaginal administration of the recommended dose; however, the rate of tachysystole was lower in the sublingual group, and this route of administration may prove a safe alternative for the induction of labor. Nevertheless, due to the small sample size in this study, further randomized studies with larger sample sizes should be conducted to confirm these findings, as well as a meta-analysis of studies using low doses of sublingual misoprostol to determine the effectiveness and safety of the method. It would be interesting to see other investigators continue with this line of research into the safety of low-dose misoprostol administered sublingually. An important aspect is women’s preference regarding the route of administration of this drug. In this study, preference was for the sublingual route, probably due to the prospect of being able to reduce the number of digital vaginal examinations required; however, at the moment, its use should be restricted to research protocols.

## Data Availability

The datasets used and/or analyzed during the current study are available from the corresponding author upon reasonable request.

## Abbreviations

95%CI: 95% confidence intervals
CDC: Centers for Disease Control and Prevention
FHR: Fetal Heart Rate
IMIP: *Instituto de Medicina Integral Prof. Fernando Figueira*
MEAC: Assis Chateaubriand Maternity Teaching Hospital
NNH: Number Needed to Harm
OR: Odds ratio
RR: Risk ratios
